# Neuroimaging of the Most Common Meningitis and Encephalitis of Adults: A Narrative Review

**DOI:** 10.3390/diagnostics14111064

**Published:** 2024-05-21

**Authors:** Teresa Perillo, Raffaella Capasso, Antonio Pinto

**Affiliations:** Department of Radiology, CTO Hospital, AORN dei Colli, 80141 Naples, Italy; dott.ssacapasso@gmail.com (R.C.); antonio.pinto@ospedalideicolli.it (A.P.)

**Keywords:** meningitis, encephalitis, magnetic resonance imaging, computed tomography

## Abstract

Meningitis is the infection of the meninges, which are connective tissue membranes covering the brain, and it most commonly affects the leptomeninges. Clinically, meningitis may present with fever, neck stiffness, altered mental status, headache, vomiting, and neurological deficits. Encephalitis is an infection of the brain, which usually presents with fever, altered mental status, neurological deficits, and seizure. Meningitis and encephalitis are serious conditions which could also coexist, with high morbidity and mortality, thus requiring prompt diagnosis and treatment. Imaging plays an important role in the clinical management of these conditions, especially Magnetic Resonance Imaging. It is indicated to exclude mimics and evaluate the presence of complications. The aim of this review is to depict imaging findings of the most common meningitis and encephalitis.

## 1. Introduction

Meningitis is the infection of the meninges, which are connective tissue membranes covering the brain [1]. They consist of three meninges, namely dura, arachnoid, and pia mater. Together, arachnoid and pia mater constitute the so-called leptomeninges. The Dura mater is the most external layer, whereas the leptomeninges are separated by the subarachnoid space, with the pia mater being adherent to the brain and rich blood supply [2]. Dura mater shows linear and thin enhancement (especially on falx and tentorium), whereas enhancement of the pia is very thin and usually not visible [3].

The most common entry routs of infectious agents into the central nervous system (CNS) are hematogenous dissemination, direct implantation (which can be traumatic or iatrogenic), local extension (for instance, from sinusitis, cellulitis, and mastoiditis), and spread through nerves [4]. Clinically, meningitis may present with fever, neck stiffness, altered mental status, headache, vomiting, and neurological neurologic deficits [5].

Imaging plays an important role in clinical management, but findings are not specific with respect to the causative pathogen [1]. Thus, cerebrospinal fluid (CSF) analysis is the diagnostic modality of choice for the identification of the responsible agent. Computed tomography (CT) should be performed in patients with impairment of consciousness and/or neurologic deficits before lumbar puncture to exclude increased intracranial pressure and brain herniations [6]. On the other hand, magnetic resonance imaging (MRI) with contrast injection is the imaging modality of choice to detect abnormal meningeal enhancement, which is present in almost 50% of patients, and it is depicted on post-contrast T1 and fluid-attenuated inversion recovery (FLAIR) weighted images [7]. MRI can also exclude mimics and evaluate the presence of complications [1]. Pathological meningeal enhancement can be pachymeningeal or leptomeningeal [1]. It is caused by the breakdown of the blood–brain barrier by inflammatory mediators released and induced by the presence of infectious agents in the CNS. On MRI, pachymeningeal enhancement appears as thickening of the dura mater, which is usually asymmetric and can be smooth or nodular. On the other hand, leptomeningeal enhancement is visible along the pial surface of the brain, filling the subarachnoid spaces, looking serpentine and frequently asymmetric [8].

Encephalitis is defined as the inflammation of the brain, which can be infectious or autoimmune [1]. Clinically, it causes fever, altered mental status, neurological deficits, and seizures. CSF analysis and MRI play a crucial role in diagnosis, which needs to be prompt as it is a serious condition, with high morbidity. Meningitis and encephalitis may also coexist due to the direct extension of the infectious process from the meninges to the brain.

The aim of this review is to depict imaging findings of the most common meningitis and encephalitis (Table 1 and Table 2).

**Table 1:** Principal imaging findings of the most common meningitis and encephalitis of the adult.

Meningitis/Encephalitis	Imaging Findings
**Piogenic Meningitis**	• Cerebrospinal fluid hyperintensity in T1 and FLAIR • Restricted diffusion of the subarachnoid spaces • Meningeal enhancement on T1 and FLAIR
**Tuberculosis**	• Leptomeningeal enhancement in the basal cisterns • Hydrocephalus very common • Infarcts in the basal ganglia due to vasculitis • Possible concomitant tuberculomas or miliary tuberculosis
**Cryptococcus Neoformans**	• Leptomeningeal enhancement • Cryptococcoma • Gelatinous pseudocysts
**Candida albicans**	• Microabscesses • Vascular lesions
**Aspergillus fumigatus**	• Abscesses • Vascular lesions
**Herpes Virus type 1**	• Mesio-temporal involvement • Bilateral and asymmetric pattern • Cortical hyperintensity on T2 and FLAIR, with restricted diffusion (acute phase) and cortical enhancement (subacute phase) • Hemorrhagic foci
**Varicella Zoster Virus**	• Leptomeningeal enhancement • Cerebellitis
**Cytomegalovirus**	• Hyperintense areas in T2 and FLAIR in the periventricular white matter • Ventriculitis
**Human herpesvirus type 6**	• Similar to herpes virus type 1, but cortical enhancement is more common
**West Nile virus**	• Hyperintensity in T2 and FLAIR of basal ganglia, thalami and midbrain
**Enterovirus**	• Rhombencephalitis

**Table 2:** Main scientific articles on which the literature review is based [1,9,10,11,12].

Essential Articles
Mohan S, Jain KK, Arabi M, Shah GV. Imaging of meningitis and ventriculitis. Neuroimaging Clin N Am. 2012.
Patkar D, Narang J, Yanamandala R, Lawande M, Shah GV. Central nervous system tuberculosis: pathophysiology and imaging findings. Neuroimaging Clin N Am. 2012
Mathur M, Johnson CE, Sze G. Fungal infections of the central nervous system. Neuroimaging Clin N Am. 2012.
Rath TJ, Hughes M, Arabi M, Shah GV. Imaging of cerebritis, encephalitis, and brain abscess. Neuroimaging Clin N Am. 2012
Abbuehl LS, Branca M, Ungureanu A, Federspiel A, Leib SL, Bassetti CLA, Hakim A, Dietmann A. Magnetic resonance imaging in acute meningoencephalitis of viral and unknown origin: Frequent findings and prognostic potential. Front Neurol. 2024

## 2. Piogenic Meningitis

Piogenic meningitis is a serious disease with high morbidity and mortality and an incidence of almost 6 cases per 100,000 adults [5]. The most common causative agent in adults is Streptococcus pneumoniae, followed by Hemophilus influenzae and Neisseria Meningitidis [13]. In immunocompromised patients, the most involved pathogens are Escherichia coli, Klebsiella, and Pseudomonas [1].

**Figure 1:**
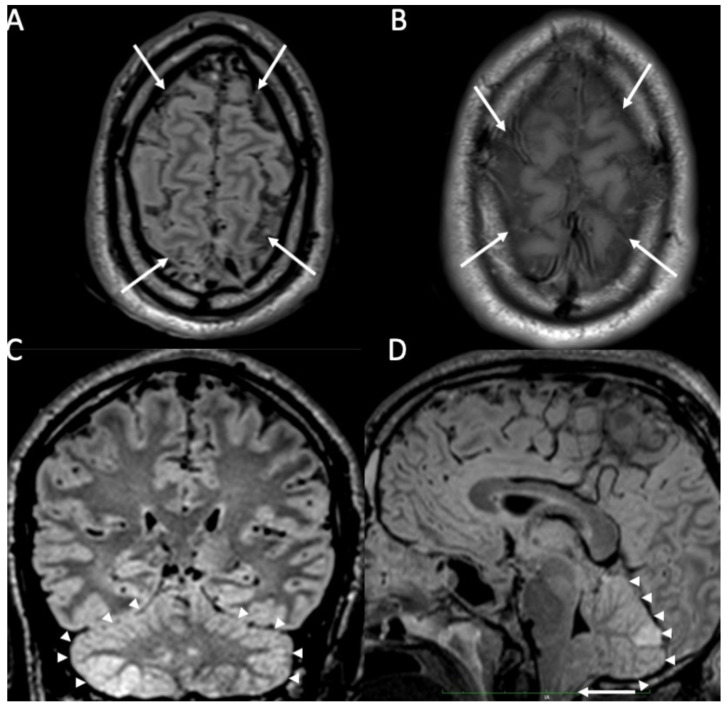
Axial FLAIR (**A**), T1 (**B**), coronal (**C**), and sagittal FLAIR (**D**) show diffuse hyperintensity on FLAIR and T1 of the subarachnoid spaces mainly in the cerebral convexity (arrows in **A**,**B**) in a patient with Streptococcus Pneumoniae meningitis. Note also concomitant cerebellitis, which appears as diffuse cortical hyperintensity on FLAIR of the cerebellum (arrowheads in **C**,**D**), with herniation of the cerebellar tonsils (arrow in **D**).

**Figure 2:**
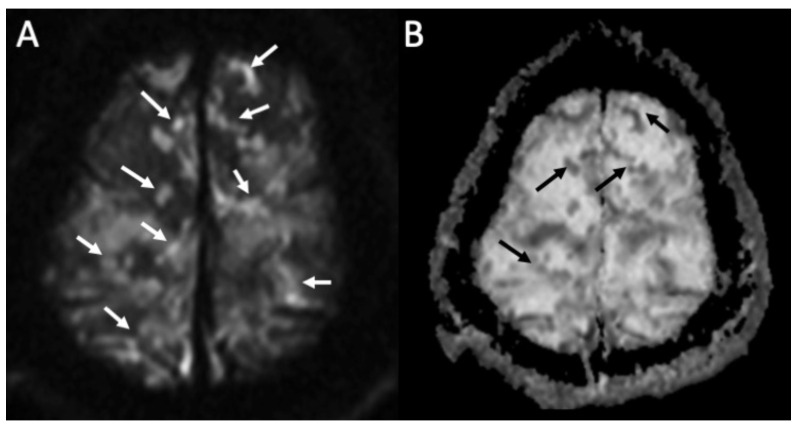
Axial DWI (**A**), ADC (**B**) shows multiple foci of restricted diffusion in the subarachnoid spaces at convexity bilaterally (arrows in **A**,**B**) in a patient with newly diagnosed Streptococcus Pneumoniae meningitis.

**Figure 3:**
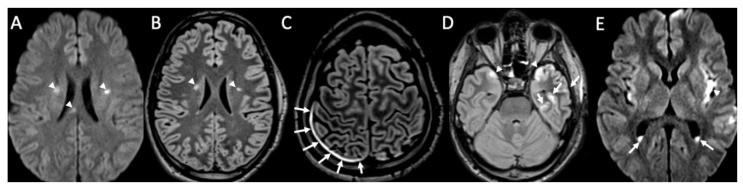
Axial DWI (**A,E**), FLAIR (**B**), enhanced FLAIR (**C**,**D**), show multiple recent ischemic areas (arrowheads in **A**,**B**,**D**) in a patient with Neisserria Meningitidis infection. Note also pachymeningeal (arrows in **C**) and leptomeningeal enhancement (arrows **D**) and purulent material in the subarachnoid spaces in the left insular region (arrowhead in **E**) and in the lateral ventricle bilaterally (arrows in **E**).

**Figure 4:**
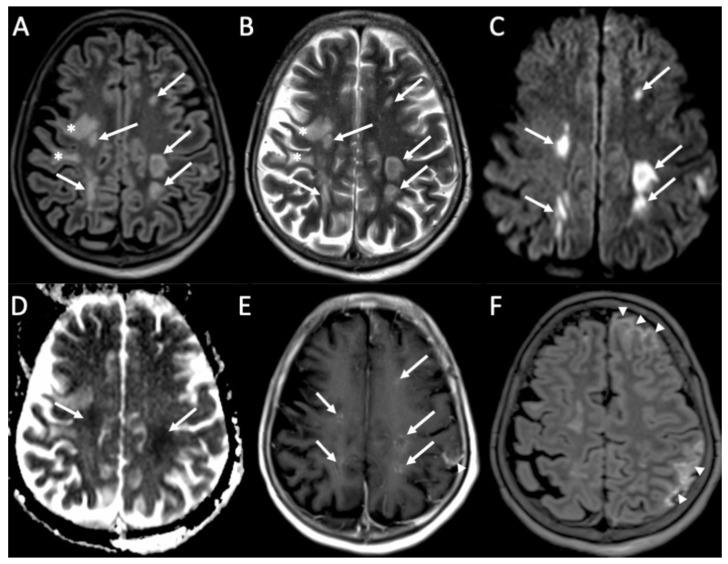
Axial FLAIR (**A**), T2 (**B**), DWI (**C**), ADC (**D**), enhanced T1 (**E**), and FLAIR (**F**) of a patient with Streptococcus Pneumoniae depict multiple recent ischemic areas in the white matter in the fronto-parietal regions bilaterally (arrows in **A**–**E**), which are hyperintense on FLAIR and T2, show restricted diffusion and faint contrast enhancement. There is also a leptomeningeal enhancement in the right frontal and parietal regions (arrowheads in **E**,**F**). Note also a gliotic area in the right frontal region (*) due to a known previous ischemic event.

MRI with contrast injection is the imaging modality of choice, and the most common finding is leptomeningeal enhancement, which is present in almost 50% of patients [14]. Unenhanced T1 and FLAIR may show obliteration of basal cisterns and CSF hyperintensity related to increased protein content (Figure 1) [15]. Diffusion-weighted imaging (DWI) is important for detecting purulent material, which shows restricted diffusion, and it is usually located in the subarachnoid spaces at the convexity (Figure 2) [16]. DWI can be the only positive sequence; thus, it should always be checked accurately [16]. Contrast-enhanced FLAIR is the most useful sequence, and it has proved to be more sensitive than contrast-enhanced T1 for the detection of meningeal enhancement, which can be pachimeningeal (Figure 3) or (more frequently) leptomeningeal (Figure 4) [17]. Thus, it is recommended to add this sequence to the imaging protocol in every suspected case of meningitis. Enhanced T1 may show hypervascularity even in the absence of meningeal enhancement, especially in the first phase of the disease (Figure 2) [18].

Sulcal hyperintensities on FLAIR can also be caused by subarachnoid hemorrhage, leptomeningeal carcinomatosis and melanosis, Moyamoya disease, supplemental oxygen, and motion artifact [1]. In the case of leptomeningeal enhancement, carcinomatosis should always be ruled out. On MRI, the latter causes thick and nodular leptomeningeal enhancement, frequently affecting the basal cisterns, whereas acute pyogenic or lymphocytic meningitis determines thin and smooth leptomeningeal enhancement [14].

**Figure 5:**
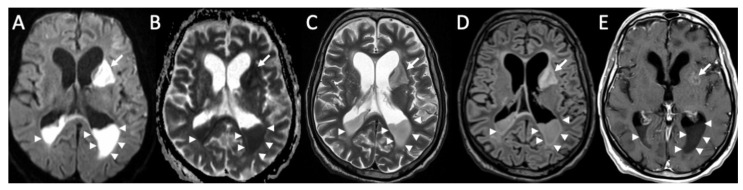
Axial DWI (**A**), ADC (**B**), T2 (**C**), FLAIR (**D**), and enhanced T1 (**E**) show purulent material in the lateral ventricles bilaterally (arrowheads in **A**–**E**), with restricted diffusion, in a patient with Streptococcus Pneumoniae meningitis. Note also the recent ischemic lesions in the left nucleo-capsular region, which show faint contrast enhancement after contrast injection (arrows in **A**–**E**). There is also enlargement of the lateral ventricles due to hydrocephalus.

Complications are quite common [19]. The most common one is hydrocephalus, which is usually mild and transient (Figure 5) [20]. It can be caused by either blockage of CSF resorption due to inflammatory debris or aqueductal obstruction [7]. It can be easily detected by both CT and MRI [1]. Rarely, ventricular dilatation may be permanent, a condition called “arrested hydrocephalus”, which does not require treatment [1,21].

**Figure 6:**
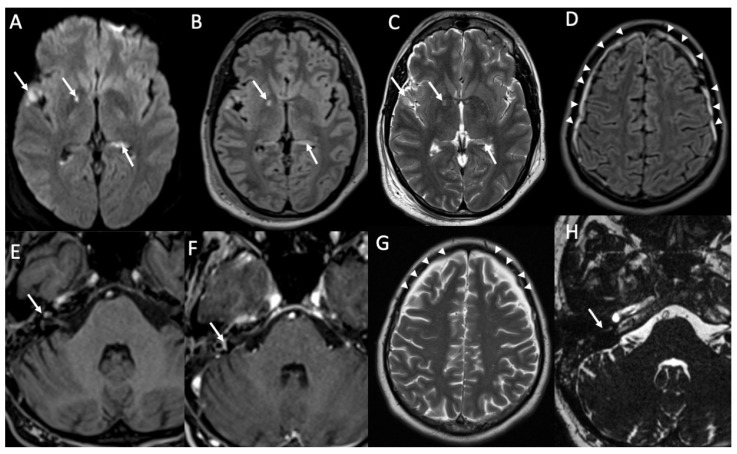
Axial DWI (**A**), FLAIR (**B**), T2 (**C**,**G**), enhanced FLAIR (**D**), T1 with fat-suppression (**E**), enhanced T1 with fat-suppression (**F**) and axial 3D-CISS show multiple recent ischemic lesions (arrows in **A**–**C**) and pachymenangeal enhancement (arrowheads in **D**) in a patient with Streptococcus pneumoniae meningitis. There is also cochlear hemorrhage on the right side (arrow in **E**), with enhancement of the structures of the inner ear (arrow in **F**). Axial T2 (**G**) and 3D-CISS (**H**) performed after one month depict subdural hygroma in the frontal regions bilaterally (arrowheads in **G**) and loss of the normal fluid signal of the internal right ear due to ossificans labyrinthitis (arrow in **H**).

Subdural effusion is present in almost 33% of patients with meningitis, and it is more frequent in pneumococcal infection (Figure 6) [19]. It is related to irritation of the dura mater or inflammation of subdural veins [22]. They are usually located at the frontal and temporal convexities and resolve spontaneously [1]. On MRI, they appear as crescentic subdural fluid collections isointense to CSF [23].

Empyema is a collection of purulent material, which can be epidural or subdural [1]. It is rarer than subdural effusion and needs to be drained surgically [7]. Epidural empyema usually has a more benign course as the dura acts as a barrier [1]. On CT, they appear as extra-axial fluid collection, which is slightly hyperdense than CSF [1]. On MRI, they are hyperintense on T1 and FLAIR, showing restricted diffusion and enhancement of the fibrous capsule after contrast injection [1].

Abscesses rarely form, especially in advanced cases [1]. On CT, they appear as round lesions with a well-defined enhancing capsule and hypodense content, surrounded by vasogenic edema [24]. On MRI, it has a well-defined capsule that shows the so-called “dual rim sign”, which is found in almost 75% of cases on T2 and susceptibility-weighted imaging (SWI) sequences [25]. It consists of two concentric rims, the outer one relatively hypointense (due to the presence of free radicals) and an inner one hyperintense (corresponding to the fibrous capsule). The purulent fluid content is hypointense on T1-weighted sequences, hyperintense on T2 and FLAIR, and shows restricted diffusion. After contrast injection, the capsule has intense and homogeneous enhancement [26]. On perfusion MRI, abscesses do not show increased cerebral blood perfusion, and this finding, together with the “dual rim sign”, is useful to distinguish them from high-grade Glioma [27]. Spectroscopy-MRI shows increased peaks of acetate, succinate, lactate, and amino acids, whereas in normal brains, these metabolites are absent [28].

Cranial nerve dysfunction is usually caused by direct inflammation, and the vestibulocochlear nerves are the most frequently involved [29]. Sensorineural hearing loss is quite common in patients with meningitis, affecting almost 30% of them, and it is usually bilateral and permanent [1]. Frequently, there is concomitant involvement of inner ear structures, which show enhancement after contrast injection on MRI in the acute phase (Figure 6) [29]. In the chronic phase, labyrinthitis ossificans may occur, which is seen on CT as sclerosis of inner ear structures and loss of their normal fluid signal on thin-section T2-weighted MRI sequences [30].

**Figure 7:**
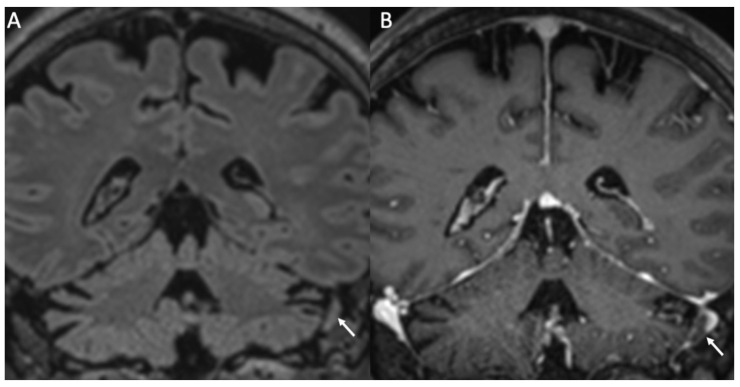
Coronal FLAIR (**A**) and enhanced T1 (**B**) show thrombosis of the right sigmoid sinus in a patient with Streptococcus pneumoniae meningitis (arrows in **A**,**B**).

Venous thrombosis is a relatively rare complication, which occurs in almost 1% of patients with meningitis [31]. On CT, thrombosed veins are spontaneously hyperdense, whereas on MRI they show loss of the normal venous signal void in unenhanced sequences and filling defects after contrast injection (Figure 7). Venous infarcts may occur, and they have a nonarterial distribution, are frequently multiple and bilateral, and have hemorrhagic areas in 25% of cases [1].

Cerebral infarcts may occur in patients with meningitis due to direct inflammation of the walls of the arteries and inflammatory-induced spasms (Figure 3, Figure 4, Figure 5 and Figure 6) [1]. They usually involve the basal ganglia.

**Figure 8:**
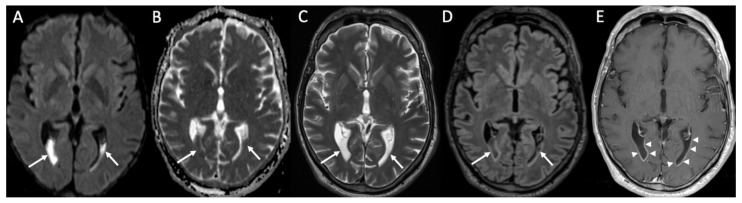
Axial DWI (**A**), ADC (**B**), T2 (**C**), FLAIR (**D**), and enhanced T1 (**E**) show purulent material in the lateral ventricles bilaterally (arrows in **A**–**D**) with linear subependymal enhancement (arrowheads in **E**) in a patient with Hemophilus influenzae meningitidis.

Ventriculitis is often associated with meningitis, probably because of the backflow of purulent material in the CSF from the extraventricular spaces to the ventricles. On MR, it appears as areas of restricted diffusion within the ventricular system, which show hyperintensity on T2, FLAIR, and T1 without contrast enhancement (Figure 3 and Figure 5) [32]. Concomitant linear subependymal enhancement of the ventricular system adjacent to the purulent material can also be present (Figure 8).

Rarely, cerebellitis may occur, especially in the pediatric population (Figure 1) [33].

## 3. Tuberculous Meningitis/Meningoencephalitis

Tuberculosis is a common infection, infecting almost one-quarter of the population worldwide, with only 10% of them developing the disease, especially in poor countries and in patients with human immunodeficiency virus (HIV) [34]. Mycobacterium tuberculosis is the most common organism causing tuberculous infection of the CNS [9]. Mycobacterium tuberculosis may reach the CNS during the initial pulmonary infection, creating the so-called “Rich focus”, which may rupture, causing meningitis.

Tuberculous meningitis is characterized by the presence of thick inflammatory exudate, which is mainly localized in the basal cisterns [9]. The exudate frequently blocks CSF flow, with subsequent hydrocephalus. It may also determine inflammation of the cranial nerves and vasculitis especially of middle cerebral and lenticulostriate arteries. On CT, enlargement of the ventricular system and infarcts may be seen, whereas on MRI, thick leptomeningeal enhancement at the basal cisterns is frequently seen, and it is highly suggestive of tuberculosis [9].

In addition, pachymeningeal lesions can be observed in tuberculosis and tend to demonstrate iso or hypointensity on T1 and T2 weighted images (this can suggest the diagnosis of a granulomatous disease) and intense and homogeneous contrast enhancement. The distribution pattern can be focal or diffuse, presenting with an “en plaque” configuration and associated with vasculitis, cranial nerve palsy, and ischemia. Restricted diffusion may be present as in other granulomatous inflammatory processes [35].

**Figure 9:**
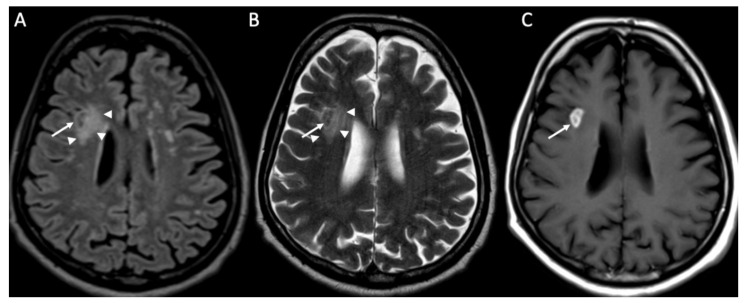
Axial FLAIR (**A**), T2 (**B**), and enhanced T1 (**C**) shows a tuberculoma in the right frontal region, which is hypointense in FLAIR and T2, and has intense contrast enhancement (arrows in **A**–**C**). Note also the surrounding vasogenic edema (arrowheads in **A**,**B**).

Concomitant granulomas may be seen, and they are called tuberculomas and they can be noncaseating or caseating [9]. Noncaseating tuberculomas on CT are iso to hypodense, whereas on MRI, they are hypointense on T1 and T2-weighted sequences, with homogeneous contrast enhancement (Figure 9). On the other hand, caseating tuberculomas are usually hyperintense on T2 images, with rim enhancement due to central liquefaction.

**Figure 10:**
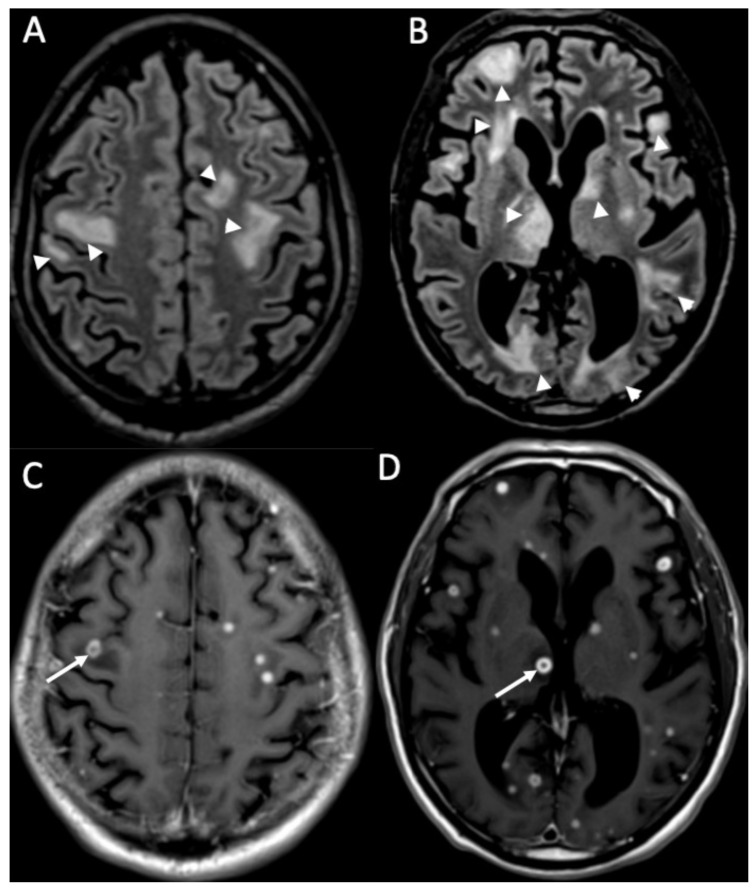
Axial FLAIR (**A**,**B**) and enhanced T1 (**C**,**D**) depict multiple tiny foci of enhancement, some of which have a ring appearance (arrows in **C**,**D**) in a patient with miliary tuberculosis. They are surrounded by vasogenic edema (arrowheads in **A**,**B**).

Miliary tuberculomas are frequently seen in patients with tuberculous meningitis [9]. It is characterized by the presence of multiple tiny lesions (with a diameter ranging from 2 to 5 millimeters), whose signal is hypointense on T2, show homogeneous contrast enhancement, and are frequently surrounded by vasogenic edema (Figure 10).

MRI spectroscopy and MRI perfusion may help distinguish tuberculomas from metastasis and gliomas, as the first shows a lipid peak and a low relative cerebral blood flow (rCBV) [36]. However, tuberculosis can also have high rCBV, so, recognizing these neuroimaging patterns on conventional MRI (T2, FLAIR, T1 pre and after contrast) is essential to obtain the correct diagnosis.

Although not routinely used, pre- and post-contrast magnetization transfer T1 images have proved useful in diagnosing CNS manifestations of tuberculosis, especially in early phases of meningitis and tuberculomas [37]. The high lipid content of tuberculous bacilli determines a decrease in the magnetization transfer ratio when compared to fungal and pyogenic meningitis and increase compared to viral meningitis.

## 4. Fungal Meningitis

Fungi are ubiquitous and do not usually cause disease in healthy individuals. In immunodepression (after pulmonary disease) and directly in the case of neurosurgical procedures or sinus disease infection, fungi may penetrate the blood–brain barrier, resulting in infection of the CNS [38]. Fungi most commonly involved in neuroinfections are Cryptococcus neoformans, Candida, and Aspergillus [39].

**Figure 11:**
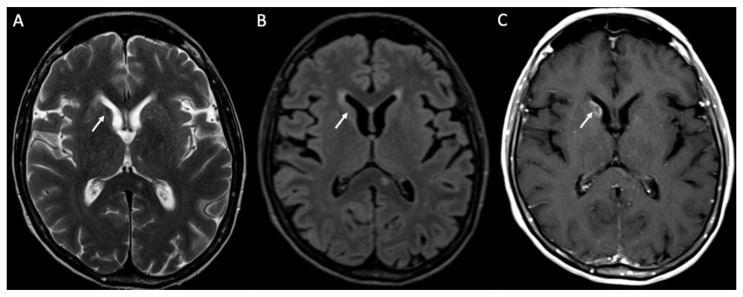
Axial T2 (**A**), FLAIR (**B**), and enhanced T1 (**C**) show a cryptococcoma in the head of the right caudate nucleus, which is slightly hypointense on T2 and FLAIR, and show inhomogeneous enhancement (arrows in **A**–**C**).

Cryptococcus neoformans (CN) is present in bird feces in the soil, and it usually causes infection in immunocompromised patients, especially with acquired immune deficiency syndrome (AIDS) [40]. CN tends to infect CNS, probably because the soluble anticryptococcal factors are absent in the CSF [41]. Frequently, imaging findings are unremarkable [10]. When present, the most common findings on MRI are leptomeningeal enhancement in the base of the brain and chronic granulomas, which are called cryptococcomas. They are located in the basal ganglia, and imaging characteristics are similar to tuberculomas (Figure 11). CN infection may spread along perivascular spaces, causing gelatinous pseudocysts, which are bilateral, symmetric, and isointense to CSF [37]. They are located in the midbrain and basal ganglia and do not show mass effect [37].

Candidiasis is usually caused by Candida albicans, which is a component of the human flora and may determine CNS disease in immunocompromised patients [42]. Neurocandidiasis may cause microabscesses, whose diameter is inferior to 3 mm. On CT, they appear iso- to hypodense, with strong contrast enhancement [10]. On MRI, their signal is variable on T2 and T1, but they show restricted diffusion and strong enhancement. Vascular involvement is present in almost 20% of patients and determines mycotic aneurysm and hemorrhage in the basal ganglia. Rarely, Candida albicans may also cause meningitis and macroabscesses.

**Figure 12:**
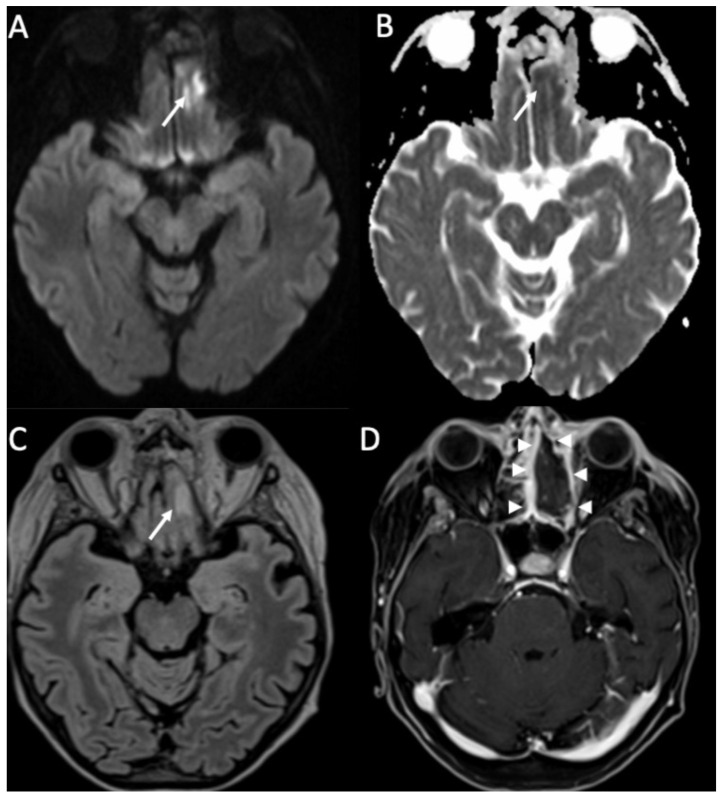
Axial DWI (**A**), ADC (**B**), FLAIR (**C**), and enhanced T1 with fat-suppression depict an area of cerebritis in the left rectus gyrus (arrows in **A**–**C**), which shows restricted diffusion and hyperintensity on FLAIR (arrow in **C**). Also note the sinusitis in the left anterior ethmoidal cells, with necrosis of the mucosa (arrowheads in **D**).

Aspergillus is commonly present in soil and water, and Aspergillus fumigatus is the most commonly involved in neuroinfection, which is seen in patients with immunodepression [43]. Aspergillus may cause cerebritis (Figure 12), which can evolve into abscesses. They are characteristically surrounded by a hypointense rim in T2 and gradient-echo images due to hemorrhage and hyphae [10]. They also show restricted diffusion peripherally. Vascular invasion is common, with vasculitis, infarction, mycotic aneurysms, and intracranial hemorrhage [44].

Mucormicosis is far less common than Cryptococcosis, Candidiasis, and Aspergillosis, and it is typically found in diabetic patients [45]. Imaging findings are similar to aspergillosis, with a high tendency to vascular invasion due to the production of elastases, which damage vascular walls [10].

## 5. Viral Meningitis and Encephalitis

Viruses may cause meningitis and/or encephalitis. Lymphocytic meningitis is commonly caused by viruses (especially enterovirus and mumps virus) and has a benign course [1]. Clinical presentation and imaging are similar to that of acute pyogenic meningitis but milder.

Viral encephalitis is the inflammation of the brain caused by a virus, and it is the most common type of encephalitis (almost 70% of the cases) [46]. Concomitant meningitis is frequent. Incidence is about 2.5–7.5 cases per 100,000 in the United States, where the most common causing viruses are herpes simplex virus (HSV), West Nile virus (WNV), Enterovirus, varicella zoster virus (VZV), cytomegalovirus (CMV) and human herpesvirus type 6 (HHV-6).

The herpes virus family is a large group of DNA viruses, which include HSV, VZV, CMV, and HHV-6. Most parts of the encephalitis are caused by HSV type 1 (HSV-1) [47]. HSV-1 encephalitis (HSE) mainly affects children and the elderly, without sex predilection, and has high mortality (almost 70%). Primary HSV-1 infection occurs with direct contact with mucosa and manifests with oral lesions, which can have a remitting-relapsing course [48]. After the primary infection, HSV-1 may be latent for years in neuronal ganglia (especially the trigeminal one) and reactivate when debilitating conditions occur. Almost 70% of HSE is related to the reactivation of HSV-1. Clinically, the triad of altered consciousness, fever, and headache is typical but not always present [48]. Seizures are also quite common.

**Figure 13:**
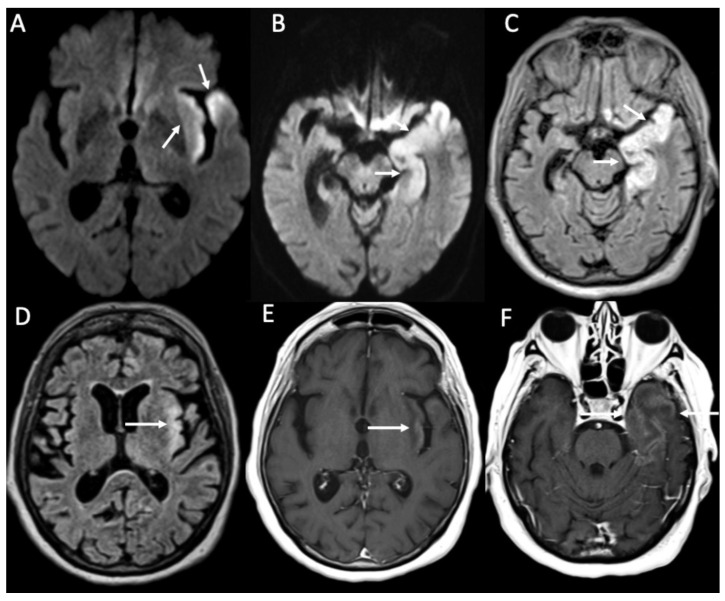
Axial DWI (**A**,**B**), FLAIR (**C**,**D**), and enhanced T1 (**E**,**F**) show fronto-temporo-insular signal alterations due to Herpes virus simplex type 1 encephalitis, which determines restricted diffusion (arrows in **A**,**B**), hyperintensity on FLAIR (arrows in **C**,**D**) and gyriform contrast enhancement (arrows in **E**,**F**).

**Figure 14:**
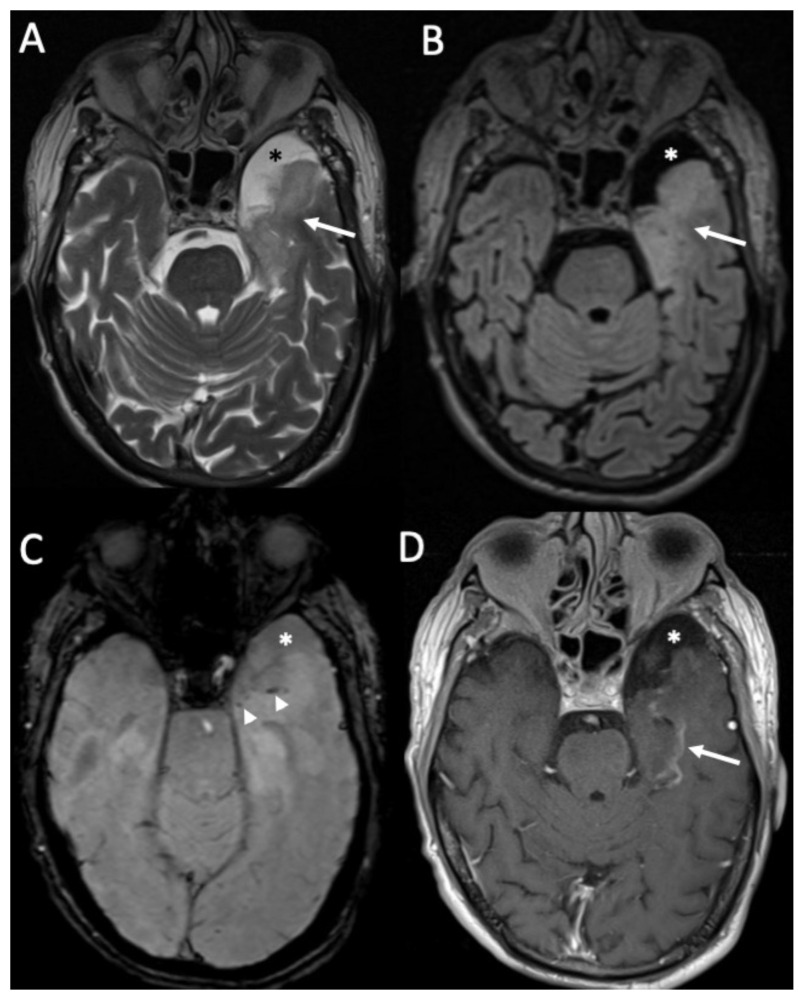
Axial T2 (**A**), FLAIR (**B**), susceptibility-weighted imaging (**C**), and enhanced T1 (**D**) show hyperintensity of the temporal lobe on T2 and FLAIR (arrows in **A**,**B**) in a patient with Herpes virus simplex type 1 encephalitis in the subacute phase. There are also some hemorrhagic foci (arrowheads in **C**) and contrast enhancement (arrow **D**). Note also concomitant temporo-polar arachnoid cyst (asterisk in **A**–**D**).

**Figure 15:**
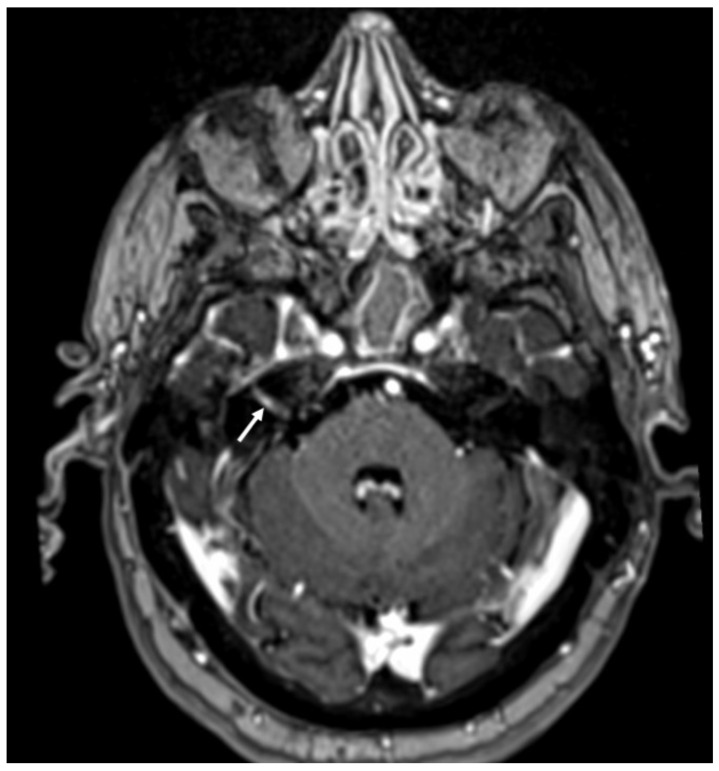
Enhanced T1 shows enhancement of the right VII-VIII nerves (arrow) in a patient with Herpes virus simplex in the cerebrospinal fluid.

In HSE, involvement of the temporal lobe and limbic system is typical, and it is probably related to the intracranial spread of HSV-1 through meningeal branches of the trigeminal ganglion [49]. In the early stages, CT can be unremarkable [50]. Later, it may show hypodensity in the temporal and insular cortex and cingulate gyrus, which is bilateral and asymmetric. On MRI, there is restricted diffusion of the cortex in the same regions, which appear hypointense on T1 and hyperintense on T2 and FLAIR sequences (Figure 13) [51]. In the subacute phase, there is swelling of the involved areas, and leptomeningeal and/or parenchymal enhancement may occur as well as hemorrhagic foci (Figure 14) [25]. Rarely can HSV-1 determine sudden hearing loss without neuroimaging findings of encephalitis. On MRI, the only finding is enhancement of the acoustic nerves (Figure 15) [52].

**Figure 16:**
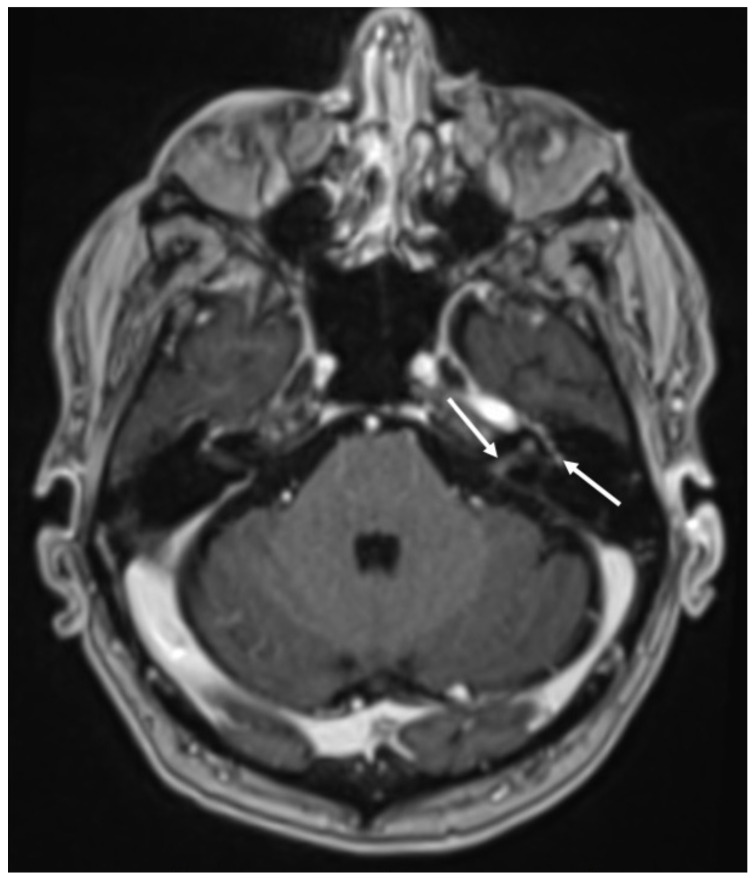
Axial enhanced T1 shows enhancement of the left acoustic nerve and homolateral facial nerve (arrows) in a patient with Ramsay–Hunt syndrome.

**Figure 17:**
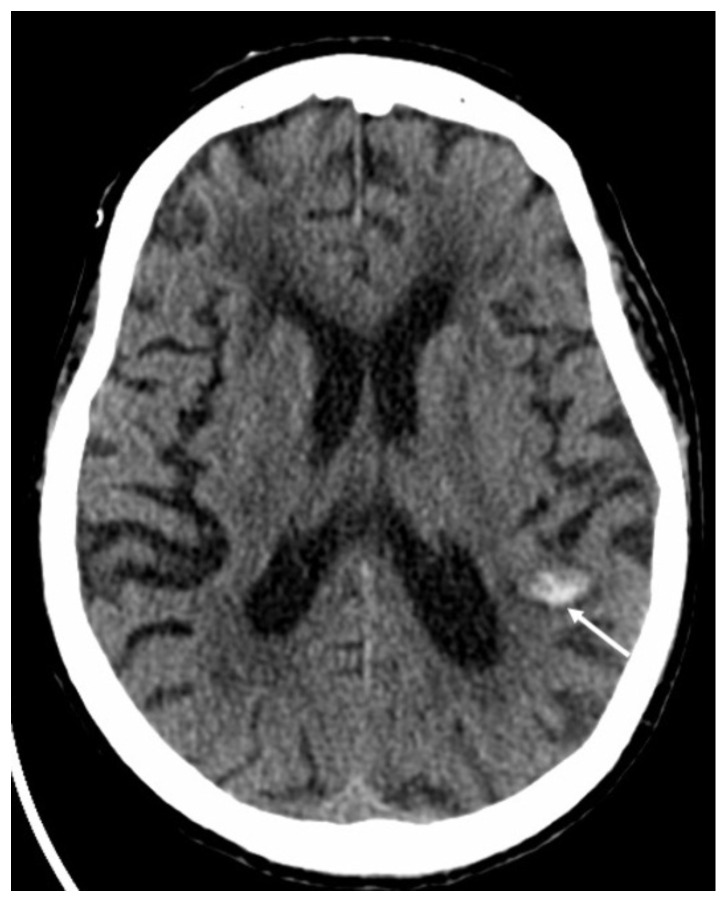
Axial computed tomography shows a small hemorrhage in the subcortical region of the left parietal region (arrow) in a patient with varicella zoster virus encephalitis.

VZV may determine varicella and herpes zoster, and transmission requires close contact between individuals [53]. After primary infection, the virus may be latent for years in nerve ganglia (especially the trigeminal) and reactivate after years, usually in the elderly or in case of debilitating conditions [54]. Meningitis is the most common CNS complication of VZV, but it can also present with Ramsay–Hunt syndrome (RHS), Herpes-zoster ophthalmicus (HZO), and focal cerebral arteriopathy (FCA). VZV meningitis imaging is that of lymphocytic meningitis and may be associated with cerebellitis, which is better evaluated with MRI and appears as diffuse cortical signal alteration, which is hypointense on T1, hyperintense on T2 and FLAIR sequences, and may show enhancement and cortical swelling, especially in advanced phases [55]. Clinically, RHS involves the geniculate ganglion, with VII and VIII cranial nerve palsy. CT may be unremarkable, whereas MRI shows abnormal enhancement of these nerves, which is linear and smooth (Figure 16) [56]. Rarely, on MRI, signal alterations in the trigeminal nucleus in the brainstem may be found, which appear hyperintense on T2 and FLAIR and show contrast enhancement [57]. In HZO, there may be extensive involvement of the optic system, including optic nerves and chiasm, which appear hyperintense on T2 and FLAIR sequences and may show contrast enhancement [58]. FCA is a late complication that mainly affects the pediatric population and immunocompromised adults [59]. It affects proximal anterior circulation, and in particular of middle cerebral arteries, which may show intense wall enhancement (detectable on vessell-wall imaging), with small infarcts (Figure 17) in the lenticulostriate territory [60].

CMV encephalitis may occur in immunocompromised patients, presenting with altered mental status, impaired memory, and cognition decline [61]. On MRI, white matter hyperintensity on T2 and FLAIR may be present, especially in periventricular areas, without enhancement [62]. Periventricular linear or punctiform areas of restricted diffusion in the periventricular white matter may be found, and they are highly suggestive of CMV infection [63]. Ventriculitis may also be present, with ependymal enhancement and hydrocephalus [64].

HHV-6 is a DNA virus of the Herpesviridae family, which causes roseola infantum and exanthema subitum in children [65]. After a primary infection, which is often asymptomatic, HHV-6 remains silent for years and may reactivate in case of immunodepression, especially related to bone marrow transplantation [66]. Imaging findings are similar to HSE, but since treatment is different, it is important to distinguish these two entities [67]. On CT, there is usually parenchymal swelling, cortical hypodensity, and enhancement in mesio-temporal regions [68]. In the early phase, MRI enables the detection of subtle signal alterations in hippocampi and amygdala, which appear as hypointensity on T1 and hyperintensity in T2 and FLAIR. Signal alterations can also be present in the insula, frontal base, parieto-occipital lobes, and deep white matter, thus resembling HSV-1 distribution [68]. Restricted diffusion is also common. In the subacute phase, cortical enhancement is frequent, and it is more common than HSE.

West Nile virus (VNV) is a flavivirus which is usually transmitted by mosquito bite and is most common in North Africa and East and Middle Asia [69]. Only 20% of infected patients become symptomatic, presenting with fever, headache, arthralgia, vomiting, diarrhea, and rash [70]. Rarely, patients may develop encephalitis and/or meningitis. Imaging findings are not specific, and on MRI, the most common finding is hyperintensity on T2 and FLAIR of the basal ganglia, thalami, midbrain, and, more rarely, temporal mesial structures. Restricted diffusion of these areas without signal alteration has also been described [71]. Contrast enhancement is rare.

Enteroviruses are a large group of RNA viruses of the picornavirus family [72]. EV68 and EV71 are the most commonly involved in encephalitis, and clinically, they present first with hand-foot-and-mouth disease and then with aseptic meningitis [73]. On MRI, rhombencephalitis is the most common finding, with frequent involvement of the posterior aspect of the midbrain, which appears to be hyperintense on T2 and FLAIR sequences. Clinically, patients present with ataxia, nystagmus, oculomotor palsies, or bulbar palsy [74]. Spinal cord lesions can also be found, especially in the cervical spine.

## 6. Conclusions

Meningitis and encephalitis are common and serious diseases which require prompt diagnosis and treatment. Neuroimaging plays a crucial role in diagnosis and management. Therefore, it is important for the neuroradiologist to be familiar with the most common entities of these conditions to guide diagnosis.
